# How To Optimally Combine Genotypic and Phenotypic Drug Susceptibility Testing Methods for Pyrazinamide

**DOI:** 10.1128/AAC.01003-20

**Published:** 2020-08-20

**Authors:** Claudio U. Köser, Daniela M. Cirillo, Paolo Miotto

**Affiliations:** a Department of Genetics, University of Cambridge, Cambridge, United Kingdom; b Emerging Bacterial Pathogens Unit, IRCCS Ospedale San Raffaele, Milan, Italy

**Keywords:** genotypic DST, *pncA*, pyrazinamide

## Abstract

False-susceptible phenotypic drug-susceptibility testing (DST) results for pyrazinamide due to mutations with MICs close to the critical concentration (CC) confound the classification of *pncA* resistance mutations, leading to an underestimate of the specificity of genotypic DST. This could be minimized by basing treatment decisions on well-understood mutations and by adopting an area of technical uncertainty for phenotypic DST rather than only testing the CC, as is current practice for the Mycobacterium tuberculosis complex.

## INTRODUCTION

Pyrazinamide (PZA) is critical for the treatment of tuberculosis (TB) (1). Because the Bactec MGIT system has a higher random rate of false resistance to PZA than to other drugs, phenotypic drug-susceptibility testing (pDST) is not carried out at all in many countries with a high incidence of TB (2, 3). Instead, WHO has concluded that *pncA* sequencing may be the most reliable method for ruling in PZA resistance (2). Because several targeted next-generation sequencing assays are being developed for direct testing of clinical samples and may be used as reflex tests for resistant cases diagnosed with point-of-care assays, *pncA* sequencing may soon become routine even in high-incidence settings (4, 5). In this scenario, the question becomes how to interpret these sequencing results and whether pDST is still needed. This is particularly challenging given that *pncA* is a nonessential gene, and there is no strong selection for particular resistance mutations, which means that a large spectrum of resistance variants is possible (e.g., 3,740 single nonsynonymous changes [6]).

We propose five groups of *pncA* mutations to inform the use of PZA and the role of additional pDST (Table 1). Group A comprises variants for which sufficient evidence exists to confidently classify them as associated with resistance and assumed to be causative of resistance. Group E encompasses mutations that are confidently not associated with resistance (i.e., neutral) (7). Routine pDST would not be needed to refine the classification of mutations in these groups. Mutations in groups B and D are likely only associated with resistance and likely neutral, respectively (i.e., additional evidence is needed before they can be moved to group A or E). Finally, group C is reserved for variants for which insufficient evidence exists.

**TABLE 1:**
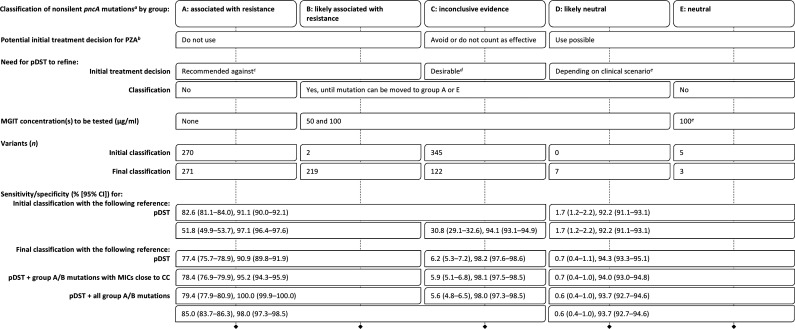
Performance characteristics of nonsilent *pncA* mutations and proposal for clinical decision making and additional pDST

a This proposal assumes the use of a high-quality sequencing technology and analysis pipeline with a negligible false-positive rate. All gDST results must be interpreted considering the likely PPV in light of the prevalence of PZA resistance for the strain in question (e.g., otherwise pan-susceptible strains versus MDR strains [29]). If there is a concern regarding the PPV of a *pncA* result because of the low prevalence of PZA resistance, resequencing would be the appropriate initial test to exclude a random sequencing error.

b The regimen may play a role (e.g., some researchers do not consider PZA resistance to be an exclusion criterion for the standardized shorter MDR regimen, despite the current World Health Organization guidelines [30–32]).

c Because of the prior evidence that links group A and B mutations with resistance, we recommend not to use PZA for MICs of >50 μg/ml. We even caution against relying on a single MIC of ≤50 μg/ml because of random errors (e.g., laboratory errors). In contrast, if multiple MICs suggest that a group A or B mutation may not be classified correctly, then these findings would have to be reviewed together with the data that underpinned the original classification to revise it if warranted. Additional types of data, such as those from the Wayne assay, may be needed in this context.

d For an MIC of ≤50 μg/ml, PZA could be used and counted as effective; for 100 μg/ml, the result is uncertain (i.e., because cutoff errors are possible for *pncA* mutations), and we recommend to continue to avoid PZA or to use it without counting it as effective; an MIC of >100 μg/ml would be an exclusion criterion for use. Where resources are limited and not all group C mutations can be tested, locally frequent mutations should be prioritized to rapidly identify mutations that are neutral and may, consequently, result in a poor PPV in that setting. If sequencing results were shared in real time, the burden of testing could be shared between countries with low and high incidence and coordinated between laboratories (e.g., different laboratories could be encouraged to test different strains with the same mutation to minimize bias, which we were not able to control for in this review).

e To detect resistance due to other mechanisms. It is not clear whether some mutations in other known resistance genes (e.g., *panD* or *rpsA*), let alone the yet-unknown mechanisms, also confer MICs close to the CC that would warrant testing 50 and 100 μg/ml for strains with group E mutations (the same consideration applies to strains that harbor no or only synonymous mutations). Until this question is clarified, we propose, if pDST is done at all, to test only the CC to minimize the misclassification of truly susceptible strains as uncertain (i.e., particularly in strains that are otherwise pan-susceptible and are unlikely to be monoresistant to PZA, with the exception of Mycobacterium canettii and most Mycobacterium bovis strains [29, 33–35]).

In 2017, we published a systematic review that introduced a statistical approach using so-called interpretative best-confidence values (iBCVs) that relied on likelihood ratios to classify mutations based on categorical pDST data at the critical concentration (CC) or results of the Wayne assay (6). We set out to explore the limitations of our original approach in light of the most important studies in this area. In particular, we aimed to increase the limited sensitivity by including types of data that were beyond the scope of the original review (e.g., results from engineered strains, quantitative pDST results, and interpretative approaches based on alternative statistical methods) and six expert rules (see Supplementary methods in the supplemental material) (3, 8–14).

Using this approach, the sensitivity of just 52% (95% confidence interval [CI], 50% to 54%) for group A and B mutations from the initial classification increased to 77% (95% CI, 76% to 79%) for the final classification (Table 1). This came at an apparent decrease in specificity from 97% (95% CI, 96% to 98%) to 91% (95% CI, 90% to 92%). However, we believe that the latter figure is an underestimate of the true specificity.

First and foremost, 50% (95% CI, 44% to 56%) of the 257 phenotypically susceptible strains with group A or B mutations harbored 1 of 18 mutations with MICs that were likely close to the CC given that they displayed poor reproducibility for pDST (i.e., cutoff errors). Notably, 55% (95% CI, 46% to 64%) of the 128 strains had 1 of 2 mutations that were not associated with resistance based on their iBCV (i.e., group E mutations in the initial classification [see Supplementary methods]). Among them was *pncA* T47A, which is known to have arisen subsequent to the acquisition of isoniazid and rifampin resistance in the progenitor of the Beijing-W clone responsible for a multidrug-resistant (MDR) TB outbreak in New York in the 1990s (15). A total of 82 results were available for this mutation, which was resistant in only 30% (95% CI, 21% to 42%) of cases. This suggested that the mode of the MIC distribution for this mutation is likely identical to or slightly below the CC, which is in line with the experimentally determined MICs (3, 8, 16). I31T, the second group E mutation in the initial classification, was resistant in 38% (95% CI, 18% to 62%) of cases. In accordance with the fourth expert rule that even modest MIC should be considered clinically relevant, it was not only logical to upgrade these 18 mutations to group A or B but also to consider all 128 pDST results to be false susceptible. This increased the specificity to 95% (95% CI, 94% to 96%) (Table 1).

In fact, it is plausible that most if not all group A and B mutations are true markers of resistance given that 14% (95% CI, 8% to 21%) of the 129 remaining strains tested phenotypically susceptible despite having a loss-of-function (LoF) mutation, contrary to the second expert rule. Assuming that these are false-susceptible results, as opposed to sequencing errors (i.e., using a composite reference of pDST and all group A and B mutations), this would increase the specificity to 100% with an associated sensitivity of 79% (95% CI, 78% to 81%) (Table 1).

Because of the possibility of cutoff errors (i.e., that mutations are misclassified as neutral if only few pDST results are available), only 11 mutations met the criteria for group D or E. It is, therefore, possible that some of the group C mutations are neutral. Nevertheless, including all 122 group C mutations would increase the sensitivity to 85% (95% CI, 84% to 86%) while reducing the specificity only marginally to 98% (95% CI, 97% to 99%) (Table 1). This supports earlier findings that the vast majority of nonsynonymous mutations in *pncA* cause resistance (1, 17).

In summary, false resistance, alternative resistance mechanisms, and low-frequency *pncA* mutations that are missed by standard Sanger sequencing have all been recognized as challenges for DST for PZA (17, 18). In contrast, false-susceptible results due to cutoff errors are understood less well. This is likely because laboratories in low-incidence settings that routinely conduct pDST for PZA usually do not encounter the same mutation sufficiently often to notice this phenomenon (the Beijing-W outbreak in New York is a notable exception). To minimize this risk, we propose two measures that would have to be tested in larger retrospective and prospective studies.

First, any new mutation within 40 bp upstream of *pncA* or nonsilent coding mutation that does not already meet the criteria for one of the remaining groups (e.g., LoF mutations) could be classified as a group C mutation and assumed to confer PZA resistance until disproven (i.e., PZA could either be avoided or used but not counted as effective). In effect, this would be similar to the recommendation by WHO to infer resistance to other drugs using targeted genotypic DST (gDST) assays when they do not detect a specific resistance mutation (e.g., when a wild-type probe for line probe assays [LPAs] does not bind [19, 20]). As is the case with resistance-inferred results with LPAs, this policy for *pncA* will result in poor positive predictive values (PPVs) in settings where strains with a neutral nonsilent mutation are frequent. This could be minimized by monitoring the frequencies of mutations and prioritizing pDST for dominant mutations when resources are limited (Table 1).

As a second measure, we propose that a CC of 100 μg/ml could be adopted as an area of technical uncertainty (ATU), as defined by the European Committee on Antimicrobial Susceptibility Testing (EUCAST), by testing 50 μg/ml in addition to 100 μg/ml (2, 21, 22). MICs of ≤50 μg/ml may be interpreted as susceptible, 100 μg/ml as uncertain, and >100 μg/ml as resistant, depending on the *pncA* mutation (Table 1).

We note, however, that these proposals rest on two assumptions. First, it is not clear whether the current CC of 100 μg/ml actually corresponds to the epidemiological cutoff value (ECOFF) (8). Rather than addressing this question using the current MGIT protocol, we recommend that, pending further head-to-head comparisons, one of the protocols that have been shown to reduce the random false resistance rate be adopted as the standard protocol for MGIT testing for PZA, which would be used to rigorously define the ECOFF (3, 23–25). Indeed, it is possible that an optimized MGIT protocol may reduce the degree of overlap between MIC distributions and, therefore, the need for an ATU, as recently proposed for rifampin (26). Second, the current CC is used as a clinical breakpoint, as defined by EUCAST, even though pharmacokinetic/pharmacodynamic and clinical data have not been systematically assessed (e.g., it is possible that the current dose of PZA is not optimal even for strains that do not have elevated MICs or that a higher dose may compensate for modest MIC increases) (27, 28).

## Supplementary Material

Supplemental file 1

Supplemental file 2

## References

[1] Lamont EA, Dillon NA, Baughn AD 2020 The bewildering antitubercular action of pyrazinamide. Microbiol Mol Biol Rev 84:e00070-19. doi:10.1128/MMBR.00070-19. 32132245 PMC7062198

[2] World Health Organization. 2018 Technical manual for drug susceptibility testing of medicines used in the treatment of tuberculosis. World Health Organization, Geneva, Switzerland http://apps.who.int/iris/bitstream/handle/10665/275469/9789241514842-eng.pdf.

[3] Morlock GP, Tyrrell FC, Baynham D, Escuyer VE, Green N, Kim Y, Longley-Olson PA, Parrish N, Pennington C, Tan D, Austin B, Posey JE 2017 Using reduced inoculum densities of *Mycobacterium tuberculosis* in MGIT pyrazinamide susceptibility testing to prevent false-resistant results and improve accuracy: a multicenter evaluation. Tuberc Res Treat 2017:3748163. doi:10.1155/2017/3748163. 29250443 PMC5698819

[4] El Achkar S, Demanche C, Osman M, Rafei R, Ismail MB, Yaacoub H, Pincon C, Duthoy S, De Matos F, Gaudin C, Trovato A, Cirillo DM, Hamze M, Supply P 2019 Drug-resistant tuberculosis, Lebanon, 2016–2017. Emerg Infect Dis 25:564–568. doi:10.3201/eid2503.181375. 30789124 PMC6390733

[5] Fofana MO, Dowdy DW 2017 Reply to Anthony et al., “Protecting pyrazinamide, a priority for improving outcomes in multidrug-resistant tuberculosis treatment”. Antimicrob Agents Chemother 61:e00427-17. doi:10.1128/AAC.00427-17. 28539499 PMC5444152

[6] Miotto P, Tessema B, Tagliani E, Chindelevitch L, Starks AM, Emerson C, Hanna D, Kim PS, Liwski R, Zignol M, Gilpin C, Niemann S, Denkinger CM, Fleming J, Warren RM, Crook D, Posey J, Gagneux S, Hoffner S, Rodrigues C, Comas I, Engelthaler DM, Murray M, Alland D, Rigouts L, Lange C, Dheda K, Hasan R, Ranganathan UDK, McNerney R, Ezewudo M, Cirillo DM, Schito M, Köser CU, Rodwell TC 2017 A standardised method for interpreting the association between mutations and phenotypic drug resistance in *Mycobacterium tuberculosis*. Eur Respir J 50:1701354. doi:10.1183/13993003.01354-2017. 29284687 PMC5898944

[7] Merker M, Kohl TA, Barilar I, Andres S, Fowler PW, Chryssanthou E, Ängeby K, Jureen P, Moradigaravand D, Parkhill J, Peacock SJ, Schön T, Maurer FP, Walker T, Köser C, Niemann S 2020 Phylogenetically informative mutations in genes implicated in antibiotic resistance in *Mycobacterium tuberculosis* complex. Genome Med 12:27. doi:10.1186/s13073-020-00726-5. 32143680 PMC7060619

[8] Werngren J, Sturegård E, Juréen P, Ängeby K, Hoffner S, Schön T 2012 Reevaluation of the critical concentration for drug susceptibility testing of *Mycobacterium tuberculosis* against pyrazinamide using wild-type MIC distributions and *pncA* gene sequencing. Antimicrob Agents Chemother 56:1253–1257. doi:10.1128/AAC.05894-11. 22203587 PMC3294906

[9] Miotto P, Cabibbe AM, Feuerriegel S, Casali N, Drobniewski F, Rodionova Y, Bakonyte D, Stakenas P, Pimkina E, Augustynowicz-Kopec E, Degano M, Ambrosi A, Hoffner S, Mansjo M, Werngren J, Rüsch-Gerdes S, Niemann S, Cirillo DM 2014 *Mycobacterium tuberculosis* pyrazinamide resistance determinants: a multicenter study. mBio 5:e01819-14. doi:10.1128/mBio.01819-14. 25336456 PMC4212837

[10] Walker TM, Kohl TA, Omar SV, Hedge J, Del Ojo Elias C, Bradley P, Iqbal Z, Feuerriegel S, Niehaus KE, Wilson DJ, Clifton DA, Kapatai G, Ip CL, Bowden R, Drobniewski FA, Allix-Beguec C, Gaudin C, Parkhill J, Diel R, Supply P, Crook DW, Smith EG, Walker AS, Ismail N, Niemann S, Peto TE, Modernizing Medical Microbiology Informatics Group. 2015 Whole-genome sequencing for prediction of *Mycobacterium tuberculosis* drug susceptibility and resistance: a retrospective cohort study. Lancet Infect Dis 15:1193–1202. doi:10.1016/S1473-3099(15)00062-6. 26116186 PMC4579482

[11] Whitfield MG, Warren RM, Streicher EM, Sampson SL, Sirgel FA, van Helden PD, Mercante A, Willby M, Hughes K, Birkness K, Morlock G, van Rie A, Posey JE 2015 *Mycobacterium tuberculosis pncA* polymorphisms that do not confer pyrazinamide resistance at a breakpoint concentration of 100 micrograms per milliliter in MGIT. J Clin Microbiol 53:3633–3635. doi:10.1128/JCM.01001-15. 26292310 PMC4609709

[12] Farhat MR, Sultana R, Iartchouk O, Bozeman S, Galagan J, Sisk P, Stolte C, Nebenzahl-Guimaraes H, Jacobson K, Sloutsky A, Kaur D, Posey J, Kreiswirth BN, Kurepina N, Rigouts L, Streicher EM, Victor TC, Warren RM, van Soolingen D, Murray M 2016 Genetic determinants of drug resistance in *Mycobacterium tuberculosis* and their diagnostic value. Am J Respir Crit Care Med 194:621–630. doi:10.1164/rccm.201510-2091OC. 26910495 PMC5027209

[13] Yadon AN, Maharaj K, Adamson JH, Lai YP, Sacchettini JC, Ioerger TR, Rubin EJ, Pym AS 2017 A comprehensive characterization of PncA polymorphisms that confer resistance to pyrazinamide. Nat Commun 8:588. doi:10.1038/s41467-017-00721-2. 28928454 PMC5605632

[14] Carter JJ, Walker TM, Walker AS, Whitfield MG, Morlock GP, Peto TE, Posey JE, Crook DW, Fowler PW 2019 Prediction of pyrazinamide resistance in Mycobacterium tuberculosis using structure-based machine learning approaches (version 1). bioRxiv https://www.biorxiv.org/content/10.1101/518142v2. 10.1093/jacamr/dlae037 PMC10946228 38500518

[15] Sreevatsan S, Pan X, Zhang Y, Kreiswirth BN, Musser JM 1997 Mutations associated with pyrazinamide resistance in *pncA* of *Mycobacterium tuberculosis* complex organisms. Antimicrob Agents Chemother 41:636–640. doi:10.1128/AAC.41.3.636. 9056006 PMC163764

[16] Dormandy J, Somoskovi A, Kreiswirth BN, Driscoll JR, Ashkin D, Salfinger M 2007 Discrepant results between pyrazinamide susceptibility testing by the reference BACTEC 460TB method and *pncA* DNA sequencing in patients infected with multidrug-resistant W-Beijing *Mycobacterium tuberculosis* strains. Chest 131:497–501. doi:10.1378/chest.06-1899. 17296653

[17] Werngren J, Alm E, Mansjö M 2017 Non-*pncA* gene-mutated but pyrazinamide-resistant *Mycobacterium tuberculosis*: why is that? J Clin Microbiol 55:1920–1927. doi:10.1128/JCM.02532-16. 28404681 PMC5442549

[18] Simons SO, van Ingen J, van der Laan T, Mulder A, Dekhuijzen PN, Boeree MJ, van Soolingen D 2012 Validation of *pncA* gene sequencing in combination with the mycobacterial growth indicator tube method to test susceptibility of *Mycobacterium tuberculosis* to pyrazinamide. J Clin Microbiol 50:428–434. doi:10.1128/JCM.05435-11. 22090409 PMC3264162

[19] Global Laboratory Initiative. Line probe assays for drug-resistant tuberculosis detection: interpretation and reporting guide for laboratory staff and clinicians. http://www.stoptb.org/wg/gli/assets/documents/LPA_test_web_ready.pdf. Accessed 2 November 2018.

[20] World Health Organization. A technical guidance document developed by the European Laboratory Initiative, version 1.0. https://openwho.org/courses/multi-drug-resistant-tb. Accessed 9 May 2020.

[21] EUCAST. 2019 Area of technical uncertainty (ATU) in antimicrobial susceptibility testing. http://www.eucast.org/fileadmin/src/media/PDFs/EUCAST_files/Breakpoint_tables/Area_of_Technical_Uncertainty_-_guidance_2019-1.pdf. Accessed 15 February 2020.

[22] Kahlmeter G, Giske CG, Kirn TJ, Sharp SE 2019 Point-counterpoint: differences between the European Committee on Antimicrobial Susceptibility Testing and Clinical and Laboratory Standards Institute recommendations for reporting antimicrobial susceptibility results. J Clin Microbiol 57:e01129-19. doi:10.1128/JCM.01129-19. 31315957 PMC6711922

[23] EUCAST. 2019 SOP for calibrating surrogate MIC methods for M. tuberculosis against the EUCAST reference MIC method, version 1.0. http://www.eucast.org/fileadmin/src/media/PDFs/EUCAST_files/Mycobacteria/Methods_in_AMST/CalibrationSOP_Mtb_190718.pdf. Accessed 9 September 2019.

[24] Schön T, Matuschek E, Mohamed S, Utukuri M, Heysell S, Alffenaar JW, Shin S, Martinez E, Sintchenko V, Maurer FP, Keller PM, Kahlmeter G, Köser CU 2019 Standards for MIC testing that apply to the majority of bacterial pathogens should also be enforced for *Mycobacterium tuberculosis* complex. Clin Microbiol Infect 25:403–405. doi:10.1016/j.cmi.2019.01.019. 30771527 PMC7903878

[25] Mustazzolu A, Piersimoni C, Iacobino A, Giannoni F, Chirullo B, Fattorini L 2019 Revisiting problems and solutions to decrease Mycobacterium tuberculosis pyrazinamide false resistance when using the Bactec MGIT 960 system. Ann Ist Super Sanita 55:51–54. doi:10.4415/ANN_19_01_09. 30968836

[26] Torrea G, Ng KCS, Van Deun A, Andre E, Kaisergruber J, Ssengooba W, Desmaretz C, Gabriels S, Driesen M, Diels M, Asnong S, Fissette K, Gumusboga M, Rigouts L, Affolabi D, Joloba M, De Jong BC 2019 Variable ability of rapid tests to detect *Mycobacterium tuberculosis rpoB* mutations conferring phenotypically occult rifampicin resistance. Sci Rep 9:11826. doi:10.1038/s41598-019-48401-z. 31413308 PMC6694172

[27] Kahlmeter G 2015 The 2014 Garrod Lecture: EUCAST - are we heading towards international agreement? J Antimicrob Chemother 70:2427–2439. doi:10.1093/jac/dkv145. 26089441

[28] Köser CU, Maurer FP, Kranzer K 2019 'Those who cannot remember the past are condemned to repeat it': drug-susceptibility testing for bedaquiline and delamanid. Int J Infect Dis 80S:S32–S35. doi:10.1016/j.ijid.2019.02.027. 30818049

[29] Allix-Béguec C, Arandjelovic I, Bi L, Beckert P, Bonnet M, Bradley P, Cabibbe AM, Cancino-Muñoz I, Caulfield MJ, Chaiprasert A, Cirillo DM, Clifton DA, Comas I, Crook DW, De Filippo MR, de Neeling H, Diel R, Drobniewski FA, Faksri K, Farhat MR, Fleming J, Fowler P, Fowler TA, Gao Q, Gardy J, Gascoyne-Binzi D, Gibertoni-Cruz A-L, Gil-Brusola A, Golubchik T, Gonzalo X, Grandjean L, He G, Guthrie JL, Hoosdally S, Hunt M, Iqbal Z, Ismail N, Johnston J, Khanzada FM, Khor CC, Kohl TA, Kong C, Lipworth S, Liu Q, Maphalala G, Martinez E, Mathys V, Merker M, Miotto P, Mistry N, Moore DAJ, Murray M, Niemann S, Omar SV, Ong RT-H, Peto TEA, Posey JE, Prammananan T, Pym A, Rodrigues C, Rodrigues M, Rodwell T, Rossolini GM, Sánchez Padilla E, Schito M, Shen X, Shendure J, Sintchenko V, Sloutsky A, Smith EG, Snyder M, Soetaert K, Starks AM, Supply P, Suriyapol P, Tahseen S, Tang P, Teo Y-Y, Thuong TNT, Thwaites G, Tortoli E, van Soolingen D, Walker AS, Walker TM, Wilcox M, Wilson DJ, Wyllie D, Yang Y, Zhang H, Zhao Y, Zhu B, CRyPTIC Consortium and the 100,000 Genomes Project. 2018 Prediction of susceptibility to first-line tuberculosis drugs by DNA sequencing. N Engl J Med 379:1403–1415. doi:10.1056/NEJMoa1800474. 30280646 PMC6121966

[30] World Health Organization. 2019 WHO consolidated guidelines on drug-resistant tuberculosis treatment. https://apps.who.int/iris/bitstream/handle/10665/311389/9789241550529-eng.pdf. Accessed 27 March 2019. 30946559

[31] Abidi S, Achar J, Assao Neino MM, Bang D, Benedetti A, Brode S, Campbell JR, Casas EC, Conradie F, Dravniece G, Du Cros P, Falzon D, Jaramillo E, Kuaban C, Lan Z, Lange C, Li PZ, Makhmudova M, Maug AKJ, Menzies D, Migliori GB, Miller A, Myrzaliev B, Ndjeka N, Noeske J, Parpieva N, Piubello A, Schwoebel V, Sikhondze W, Singla R, Souleymane MB, Trebucq A, Van Deun A, Viney K, Weyer K, Zhang BJ, Ahmad Khan F 2020 Standardised shorter regimens versus individualised longer regimens for rifampin- or multidrug-resistant tuberculosis. Eur Respir J 55:1901467. doi:10.1183/13993003.01467-2019. 31862767

[32] Van Deun A, Decroo T, Tahseen S, Trebucq A, Schwoebel V, Ortuno-Gutierrez N, de Jong BC, Rieder HL, Piubello A, Chiang CY 2020 World Health Organization 2018 treatment guidelines for rifampicin-resistant tuberculosis: uncertainty, potential risks and the way forward. Int J Antimicrob Agents 55:105822. doi:10.1016/j.ijantimicag.2019.10.003. 31626907

[33] Feuerriegel S, Köser CU, Richter E, Niemann S 2013 *Mycobacterium canettii* is intrinsically resistant to both pyrazinamide and pyrazinoic acid. J Antimicrob Chemother 68:1439–1440. doi:10.1093/jac/dkt042. 23447141

[34] Loiseau C, Brites D, Moser I, Coll F, Pourcel C, Robbe-Austerman S, Escuyer V, Musser KA, Peacock SJ, Feuerriegel S, Kohl TA, Niemann S, Gagneux S, Köser CU 2019 Revised interpretation of the Hain Lifescience GenoType MTBC to differentiate *Mycobacterium canettii* and members of the *Mycobacterium tuberculosis* complex. Antimicrob Agents Chemother 63:e00159-19. doi:10.1128/AAC.00159-19. 30962348 PMC6535525

[35] Loiseau C, Menardo F, Aseffa A, Hailu E, Gumi B, Ameni G, Berg S, Rigouts L, Robbe-Austerman S, Zinsstag J, Gagneux S, Brites D 2020 An African origin for *Mycobacterium bovis*. Evol Med Public Health 2020:49–59. doi:10.1093/emph/eoaa005. 32211193 PMC7081938

