# Supplementary material for: How To Optimally Combine Genotypic and Phenotypic Drug Susceptibility Testing Methods for Pyrazinamide
Source: Antimicrob Agents Chemother. 2020 Aug 20;64(9):e01003-20. doi: 10.1128/AAC.01003-20 (PMC7449218; doi:10.1128/AAC.01003-20)
Supplement: Supplemental file 1 [file AAC.01003-20-s0001.pdf]

SUPPLEMENTARY METHODS

Six expert rules

We made three assumptions in our original study (1). First, we assumed that synonymous mutations are neutral and that different nucleotide changes that result in the same amino acid change have the same effect. Second, any easily identifiable loss-of-function (LoF) mutation (e.g. nonsense mutations) should confer resistance. Third, any single *pncA* mutation that has been associated with resistance when it occurs in isolation is assumed to be necessary and sufficient to also cause resistance in other genetic backgrounds (i.e. in combination with other *pncA* mutations or mutations elsewhere in the genome).

We adopted three additional expert rules for this study. A recent meta-analysis of clinical outcome data compared with categorical pDST data found that the use of PZA is associated with significantly less treatment success and higher mortality for phenotypically resistant isolates (2). This does not mean that all isolates with MICs above the CC of 100 µg/mL have worse clinical outcomes as the association may have been largely driven by isolates with very high PZA MICs. In other words, because no pharmacokinetic/pharmacodynamic target has been endorsed by any breakpoint committee and clinical outcomes have not been correlated systematically with PZA MICs, the underlying assumption by WHO is that the current CC corresponds to a clinical breakpoint, as defined by the European Committee on Antimicrobial Susceptibility Testing, and that any MIC increase is significant (3, 4). To err on the side of caution, we assumed that even mutations with MIC distributions that are divided by the CC (i.e. that overlap with the MIC distribution of susceptible strains because some *pncA* activity is retained) are clinically relevant until evidence to the contrary is available. We also assumed that the *in vivo* resistance phenotypes can be replicated accurately using the current *in vitro* testing conditions, which may not always be the case (5). Finally, we regarded homoplasic mutations as likely signals for positive selection and, consequently, phenotypic resistance (6).

Rules for classification

<u>Initial classification: association data from Miotto et al. 2017</u>

It was not the purpose of this study to update the systematic review of categorical pDST data that resulted in Miotto et al. by rerunning the original search terms and including all studies published since the 30th December 2015 (1). Rather, we wanted to explore how the classification from Miotto et al. based on the corrected "interpretative best confidence values" (iBCVs) could be refined in light of the most important studies in this area, including those with types of data that were beyond the scope of the original review (Figure S1). We, therefore, limited this study to the 636 variants originally identified in Miotto et al. and converted them to groups A-E according to Table S1 to yield the initial classification (see below for a discussion of synonymous mutations (1)).

Table S1: Relation between iBCVs from Miotto et al. 2017 and initial classification.

LR+ v OR		Corrected iBCV	Symbol	Initial classification
***p*-value**	**value**			
<0.05	> 10	High confidence for association with resistance	●	A
<0.05	5 < … ≤ 10	Moderate confidence for association with resistance	●	
<0.05	1 < … ≤ 5	Minimal confidence for association with resistance	●	B
<0.05	< 1	No association with resistance	●	E
≥0.05	-	Indeterminate	Indeter	C

Figure S1: Overview of additional data considered to refine the initial classification.

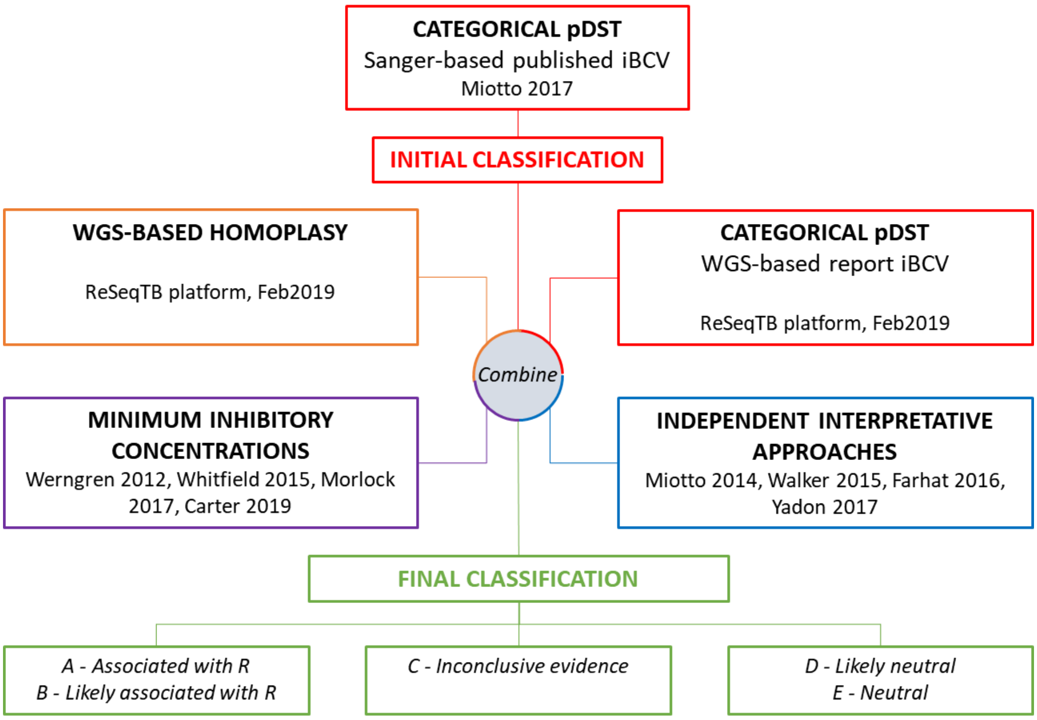

Second classification: association data from ReSeqTB

We considered additional association data from the ReSeqTB platform (ReSeqTB public platform (http://www.reseqtb.org/, PFF_2019-01-24.csv, last accessed on 4th February 2019) (7). Whenever the corrected iBCV was from this dataset was more significant than the one from Miotto et al. (e.g. C138R was originally indeterminate whereas it was a high confidence mutation in ReSeqTB), the original result was updated to yield an updated corrected iBCV and the classification was updated accordingly (i.e. based on the same logic used for the initial classification). Moreover, we upgraded all in-frame coding indels from group C to group B as these likely abolish the function of PncA. Finally, we downgraded all mutations that were more than 40 nucleotides upstream of *pncA* from group C to group D as these are unlikely to confer resistance.

Third classification: direct and indirect MIC data

Group C-E mutations in the second classification were upgraded to group B if these mutations might confer MICs close to the CC (i.e. if they were tested at least six times and yielded proportions of resistant and susceptible pDST results that were both above 25%). Moreover, all group C mutations that were tested at least five times and consistently yielded MICs≤50 µg/mL were moved to group D.

Fourth classification: homoplasy data

Homoplastic group C-E mutations in the third classification were upgraded to group B as the independent evolution of the same mutation is typically as sign of positive selection (6). Homoplasic group B mutations in the third classification that had been group B or E mutations in the second classification and had MICs close to the CC were upgraded to group A as additional categorical pDST would not be helpful for these mutations (e.g. T47A).

Final classification: additional experimental evidence

To err on the side of caution, strains with a single group A mutation in the fourth classification were downgraded to group B if these were regarded as neutral in at least one of the additional studies with experimental data or yielded inconsistent results in Yadon et al. (8). Mutations were not downgraded if their MICs were likely close to the CC (e.g. T47A). If one mutation was associated with resistance

and the other one was neutral (e.g. for S18P+P54L in Walker et al. (9)), this combination was not downgraded. If a study provided information for only one of multiple mutations, the combination of mutations was downgraded, unless one of two was a LoF mutation (e.g. W119Stop+T168I was not downgraded, even though T168I was neutral based on Walker et al. (9)).
Strains with a single group C mutation in the fourth classification were upgraded to group B if the mutation in question was associated with resistance in most studies with additional experimental evidence. The same rule was applied to strains with multiple mutations provided that these criteria were met for at least one of the mutations for each combination (e.g. g-71del+T100P was reclassified because of the evidence for T100P in Miotto et al. and Farhat et al. (1, 10)). By contrast, if a group C mutation (or all mutations in strains with multiple mutations) were consistently considered to be neutral and evidence was available from at least two studies (e.g. V45A), the mutation or combination of mutations was moved to group D.

Calculations
The performance characteristics were calculated using the Open Source Epidemiologic Statistics for Public Health (OpenEpi), Version 3.01 (www.OpenEpi.com, updated 2013/04/06, accessed 2020/04/30).

Open questions
Synonymous mutations
As part of our first expert rule, we assumed that synonymous mutations are neutral and that different nucleotide changes that result in the same amino acid change have the same effect. We found that the S65S (tcc/tcT) mutation, which is regarded as maker for a subgroup of lineage 3 (6), in one lineage 1.1.2 strain, which does not necessarily mean that this mutation is not neural. However, Yadon et al. observed several instances in which different nucleotide changes in the same codon that either result in synonymous changes or the same non-synonymous change yield different experimental results (see relevant worksheet in Supplementary Excel File (1)). If confirmed, this would mean that our expert rule does not always apply, which might be due to codon usage effects.

OVERVIEW OF "DETAILED DATASET" WORKSHEET IN SUPPLEMENTARY EXCEL FILE

Section: Categorical phenotypic drug susceptibility testing (red)

This section reports all pDST results for the genetic variants observed in *pncA* using mostly Sanger sequencing based on Supplementary Table S7.1 from Miotto et al. (1) and whole genome sequencing (WGS) from the ReSeqTB platform (http://www.reseqtb.org/, PFF_2019-01-24.csv, latest accessed on February 4th, 2019).

Miotto 2017 (PMID 29284687):

- *pncA* mutation (column A): genetic variants observed in *pncA* according to (1).
- Numbers for liquid dataset (columns E to J), Wayne's assay dataset (columns K to P) and combined dataset (column Q to V) according to (1). For each dataset:
    - Tot n of isolates (column E-K-Q): total number of isolates from (1).
    - Found in R (TP) (columns F-L-R): number of mutated Z-resistant isolates (i.e. true positives) from (1).
    - Found in S (FP) (columns G-M-S): number of mutated Z-susceptible isolates (i.e. false positives) from (1).
    - R% (Columns H-N-T): percentage of mutated isolates that are phenotypically resistant from (1) (i.e. column F/Column E %). Green = mutation is found in at least 25% of Z-resistant and susceptible cases, and the number of mutated isolates is >5.
    - S% (Column I-O-U): percentage of mutated isolates that are phenotypically susceptible from (1) (i.e. column G/Column E %). Green = mutation is found in at least 25% of Z-resistant and susceptible cases, and the number of mutated isolates is >5.
    - Corrected iMCV (columns J-P-V): Please refer to Supplementary Section 5 in (1) for more details about this interpretative medium confidence value.
- Corrected iBCV (column W): corrected iBCV according to (1).

Initial classification (column X): see Supplementary Methods.

ReSeqTB resistance report – Feb2019 sub-section

- Tot n of isolates (column Y): total number of isolates from the ReSeqTB.
- Found in R (TP) (column Z): number of mutated Z-resistant isolates (i.e. true positives) from ReSeqTB.
- Found in S (FP) (column AA): number of mutated Z-susceptible isolates (i.e. false positives) from ReSeqTB.
- R% (Column AB): percentage of mutated isolates that are phenotypically resistant from ReSeqTB (column L/Column K %).
- S% (Column AC): percentage of mutated isolates that are phenotypically susceptible from ReSeqTB (column M/Column K %).
- Corrected iBCV (column AD): corrected iBCV for ReSeqTB.
- Updated corrected iBCV (column AE): updated iBCV based on Miotto et al. (1) and ReSeqTB.

Classification 2 (column AF): see Supplementary Methods.

Section: Minimum inhibitory concentrations (purple)

- Indirect MIC data:
    - MIC likely close to CC (column AG): The MIC of a mutation can be inferred indirectly if it shows a poor reproducibility for categorical pDST (i.e. if it is tested at least six times and yields proportions of resistant and susceptible pDST results both above 25%. The relevant mutations are highlighted in green as explained above).
- Direct MIC data:

- Werngren 2012 (PMID: 22203587) (columns AH-AI): MIC data from (11).
- Whitfield 2015 (PMID 26292310) (columns AJ-AK): MIC data from (12).
- Morlock 2017 (PMID: 29250443) (column AL-AM): MIC data from (13).
- Carter 2019 (BioRxiv doi.org/10.1101/518142) (column AN-AO): MIC data from (14).
- N of isolates ≤CC (100 µg/mL) (column AP): total number of isolates with MICs below or equal to the CC of 100 µg/mL.
- N of isolates >CC (100 µg/mL) (column AQ): total number of isolates with MICs above the CC of 100 µg/mL.

- Classification 3 (column AR): see Supplementary Methods.

Section: Homoplasy (orange)

- ReSeqTB resistance report - Feb2019 (Columns AS to AU):
  - Typing information (genomes available): number of isolates with WGS.
  - Typing information (lineages): lineages assigned according to (7).
  - Homoplasy y/n: "y" shows that the mutation is homoplasic, whereas "n" indicates the mutation is not. NB: ReSeqTB accepts any WGS data, irrespective of the availability of associated pDST result. Therefore, the number of isolates with typing information may differ from the number shown in column Y.
- Classification 4 (column AV): see Supplementary Methods.

Section: Additional experimental evidence for final classification (cyan)

- Miotto 2014 (PMID: 25336456) (Column AW): This interpretation is based on a conditional inference tree model that considered results from *pncA* gene sequencing, the pyrazinamidase enzymatic activity, and PncA structure and free energy analyses (15). Premature stop codons and frameshift mutations were considered to be markers of Z-resistance by an expert rule.
- Walker 2015 (PMID: 26116186) (Column AX): The interpretation is based on a custom algorithm that was used for a training dataset and a validation dataset (9).
- Farhat 2016 (PMID: 26910495) (Column AY): The interpretation relies on random forest predictive modeling that considered results of sequencing (*pncA* and 27 additional genes) and pDST (10).
- Yadon 2017 (PMID: 28928454) (Columns AZ to BD): The interpretation is based on *in vitro* and *in vivo* selection (in BALB/c mice) of laboratory-generated mutants (8). *In vitro* (or *in vivo*) depletion corresponds to Z susceptibility, whereas *in vitro* (or *in vivo*) enrichment means Z resistance.

Section: Final classification (green)

This section reports the final classification based on all available evidence (see Supplementary Methods).

Section: For comparison (grey)

This section provides other notable classifications for comparison.

- NIPRO V1 benign mutations (Ando 2010, PMID 19832709) (column BG): The Genoscholar PZA-TB ver. I (Nipro Corporation, Japan) was designed not to detect these mutations to avoid false resistance because they were considered to be neutral (16, 17). This assay has been replaced by the Genoscholar PZA-TB ver. II kit, which is only designed to avoid false resistance due to three synonymous mutations (i.e. G60G, S65S and T142T) (18).
- Whitfield 2015 (PMID 26292310) (column BH): This interpretation is based on the association between mutations and pDST results. The study was included as it focused on defining neutral mutations (12).
- Allix-Beguec 2018 (PMID 30280646) (column BI): This interpretation is based on a specifically developed knowledge base of mutations that are predictive of resistance or consistent with susceptibility (19). The knowledge base was informed by Miotto et al. (1), a Centers for Disease Control and Prevention strain panel and two studies that had no strains in common with our study

(8, 9). This interpretation is used by Public Health England for its routine WGS-based diagnostic service (this information is current as of March 29th, 2019).

- Carter 2019 (BioRxiv doi.org/10.1101/518142) (Column BJ): This interpretation relies on a structure-based machine learning approach (14).

References

1. Miotto P, Tessema B, Tagliani E, Chindelevitch L, Starks AM, Emerson C, Hanna D, Kim PS, Liwski R, Zignol M, Gilpin C, Niemann S, Denkinger CM, Fleming J, Warren RM, Crook D, Posey J, Gagneux S, Hoffner S, Rodrigues C, Comas I, Engelthaler DM, Murray M, Alland D, Rigouts L, Lange C, Dheda K, Hasan R, Ranganathan UDK, McNerney R, Ezewudo M, Cirillo DM, Schito M, Köser CU, Rodwell TC. 2017. A standardised method for interpreting the association between mutations and phenotypic drug resistance in *Mycobacterium tuberculosis*. Eur Respir J 50:1701354.
2. Collaborative Group for the Meta-Analysis of Individual Patient Data in MDR-TB treatment, Ahmad N, Ahuja SD, Akkerman OW, Alffenaar JC, Anderson LF, Baghaei P, Bang D, Barry PM, Bastos ML, Behera D, Benedetti A, Bisson GP, Boeree MJ, Bonnet M, Brode SK, Brust JCM, Cai Y, Caumes E, Cegielski JP, Centis R, Chan PC, Chan ED, Chang KC, Charles M, Cirule A, Dalcolmo MP, D'Ambrosio L, de Vries G, Dheda K, Esmail A, Flood J, Fox GJ, Frechet-Jachym M, Fregona G, Gayoso R, Gegia M, Gler MT, Gu S, Guglielmetti L, Holtz TH, Hughes J, Isaakidis P, Jarlsberg L, Kempker RR, Keshavjee S, Khan FA, Kipiani M, Koenig SP, Koh WJ, et al. 2018. Treatment correlates of successful outcomes in pulmonary multidrug-resistant tuberculosis: an individual patient data meta-analysis. Lancet 392:821-834.
3. Kahlmeter G. 2015. The 2014 Garrod Lecture: EUCAST - are we heading towards international agreement? J Antimicrob Chemother 70:2427-39.
4. Köser CU, Maurer FP, Kranzer K. 2019. 'Those who cannot remember the past are condemned to repeat it': Drug-susceptibility testing for bedaquiline and delamanid. Int J Infect Dis 80S:S32-S35.
5. Anthony RM, den Hertog AL, van Soolingen D. 2018. 'Happy the man, who, studying nature's laws, Thro' known effects can trace the secret cause.' Do we have enough pieces to solve the pyrazinamide puzzle? J Antimicrob Chemother 73:1750-1754.
6. Merker M, Kohl TA, Barilar I, Andres S, Fowler PW, Chryssanthou E, Ängeby K, Jureen P, Moradigaravand D, Parkhill J, Peacock SJ, Schön T, Maurer FP, Walker T, Köser C, Niemann S. 2020. Phylogenetically informative mutations in genes implicated in antibiotic resistance in *Mycobacterium tuberculosis* complex. Genome Med 12:27.
7. Ezewudo M, Borens A, Chiner-Oms A, Miotto P, Chindelevitch L, Starks AM, Hanna D, Liwski R, Zignol M, Gilpin C, Niemann S, Kohl TA, Warren RM, Crook D, Gagneux S, Hoffner S, Rodrigues C, Comas I, Engelthaler DM, Alland D, Rigouts L, Lange C, Dheda K, Hasan R, McNerney R, Cirillo DM, Schito M, Rodwell TC, Posey J. 2018. Integrating standardized whole genome sequence analysis with a global *Mycobacterium tuberculosis* antibiotic resistance knowledgebase. Sci Rep 8:15382.
8. Yadon AN, Maharaj K, Adamson JH, Lai YP, Sacchettini JC, Ioerger TR, Rubin EJ, Pym AS. 2017. A comprehensive characterization of PncA polymorphisms that confer resistance to pyrazinamide. Nat Commun 8:588.
9. Walker TM, Kohl TA, Omar SV, Hedge J, Del Ojo Elias C, Bradley P, Iqbal Z, Feuerriegel S, Niehaus KE, Wilson DJ, Clifton DA, Kapatai G, Ip CL, Bowden R, Drobniewski FA, Allix-Beguec C, Gaudin C, Parkhill J, Diel R, Supply P, Crook DW, Smith EG, Walker AS, Ismail N, Niemann S, Peto TE, Modernizing Medical Microbiology Informatics G. 2015. Whole-genome sequencing for prediction of *Mycobacterium tuberculosis* drug susceptibility and resistance: a retrospective cohort study. Lancet Infect Dis 15:1193-202.
10. Farhat MR, Sultana R, Iartchouk O, Bozeman S, Galagan J, Sisk P, Stolte C, Nebenzahl-Guimaraes H, Jacobson K, Sloutsky A, Kaur D, Posey J, Kreiswirth BN, Kurepina N, Rigouts L, Streicher EM, Victor TC, Warren RM, van Soolingen D, Murray M. 2016. Genetic determinants of drug resistance in *Mycobacterium tuberculosis* and their diagnostic value. Am J Respir Crit Care Med 194:621-30.
11. Werngren J, Sturegård E, Juréen P, Ängeby K, Hoffner S, Schön T. 2012. Reevaluation of the critical concentration for drug susceptibility testing of *Mycobacterium tuberculosis* against

pyrazinamide using wild-type MIC distributions and *pncA* gene sequencing. Antimicrob Agents Chemother 56:1253-7.

12. Whitfield MG, Warren RM, Streicher EM, Sampson SL, Sirgel FA, van Helden PD, Mercante A, Willby M, Hughes K, Birkness K, Morlock G, van Rie A, Posey JE. 2015. *Mycobacterium tuberculosis pncA* polymorphisms that do not confer pyrazinamide resistance at a breakpoint concentration of 100 micrograms per milliliter in MGIT. J Clin Microbiol 53:3633-5.
13. Morlock GP, Tyrrell FC, Baynham D, Escuyer VE, Green N, Kim Y, Longley-Olson PA, Parrish N, Pennington C, Tan D, Austin B, Posey JE. 2017. Using reduced inoculum densities of *Mycobacterium tuberculosis* in MGIT pyrazinamide susceptibility testing to prevent false-resistant results and improve accuracy: a multicenter evaluation. Tuberc Res Treat 2017:3748163.
14. Carter JJ, Walker TM, Walker AS, Whitfield MG, Morlock GP, Peto TE, Posey JE, Crook DW, Fowler PW. Prediction of pyrazinamide resistance in *Mycobacterium tuberculosis* using structure-based machine learning approaches (version 1). http://doi.org/10.1101/518142.
15. Miotto P, Cabibbe AM, Feuerriegel S, Casali N, Drobniewski F, Rodionova Y, Bakonyte D, Stakenas P, Pimkina E, Augustynowicz-Kopec E, Degano M, Ambrosi A, Hoffner S, Mansjo M, Werngren J, Rüsch-Gerdes S, Niemann S, Cirillo DM. 2014. *Mycobacterium tuberculosis* pyrazinamide resistance determinants: a multicenter study. MBio 5:e01819-14.
16. Ando H, Mitarai S, Kondo Y, Suetake T, Sekiguchi JI, Kato S, Mori T, Kirikae T. 2010. Pyrazinamide resistance in multidrug-resistant *Mycobacterium tuberculosis* isolates in Japan. Clin Microbiol Infect 16:1164-8.
17. Kirikae T. Personal communication.
18. Steegen B. Personal communication.
19. CRyPTIC Consortium and the 100000 Genomes Project, Allix-Beguec C, Arandjelovic I, Bi L, Beckert P, Bonnet M, Bradley P, Cabibbe AM, Cancino-Munoz I, Caulfield MJ, Chaiprasert A, Cirillo DM, Clifton DA, Comas I, Crook DW, De Filippo MR, de Neeling H, Diel R, Drobniewski FA, Faksri K, Farhat MR, Fleming J, Fowler P, Fowler TA, Gao Q, Gardy J, Gascoyne-Binzi D, Gibertoni-Cruz AL, Gil-Brusola A, Golubchik T, Gonzalo X, Grandjean L, He G, Guthrie JL, Hoosdally S, Hunt M, Iqbal Z, Ismail N, Johnston J, Khanzada FM, Khor CC, Kohl TA, Kong C, Lipworth S, Liu Q, Maphalala G, Martinez E, Mathys V, Merker M, Miotto P, et al. 2018. Prediction of susceptibility to first-line tuberculosis drugs by DNA sequencing. N Engl J Med 379:1403-1415.
